# Carboxylated Multiwalled Carbon Nanotubes as Dispersive Solid-Phase Extraction Sorbent to Determine Eighteen Polychlorinated Biphenyls in Vegetable Samples by Gas Chromatography-Mass Spectrometry

**DOI:** 10.1155/2019/4264738

**Published:** 2019-08-20

**Authors:** Tengfei Liu, Daifeng Yang, Jian Mao, Xueming Zhang, Minghui Dong

**Affiliations:** ^1^Jiangsu Taihu Area Institute of Agricultural Sciences, Suzhou 215155, China; ^2^Suzhou Agro-Products Quality and Safety Inspection and Testing Center, Suzhou 215128, China

## Abstract

A simple, rapid, and reliable method based on dispersive solid-phase extraction (d-SPE) and gas chromatography-mass spectrometry (GC-MS) was developed for quantitating polychlorinated biphenyls (PCBs) in vegetable samples. Parameters affecting both the extraction yields and cleanup efficiency, including the type and volume of extraction solvent, extraction time, type and volume of cleanup sorbent, and cleanup time, were optimized. Matrix effects were evaluated, and matrix-matched calibration was recommended. Under the optimized conditions, carboxylated multiwalled carbon nanotubes (MWCNTs-COOH), which exhibit excellent adsorption capabilities due to large surface area and unique structure, were employed as d-SPE sorbent to remove interfering substances, rather than the analytes, from vegetable samples. Satisfactory linear relationship was observed for all PCBs across a concentration range of 5–500 *μ*g/kg with correlation coefficients no less than 0.9993. Four representative vegetables (cucumber, tomato, lettuce, and cabbage) were selected as matrices for method validation. Each matrix was spiked at concentrations of 5, 10, and 100 *μ*g/kg to evaluate recoveries, which ranged from 84.5% to 116.5% with relative standard deviations (*n*=6) between 0.6% and 17.6%. The limits of detection and the limits of quantification ranged from 0.3 to 1.4 *μ*g/kg and 0.8 to 4.5 *μ*g/kg, respectively. Twelve real vegetable samples were analyzed using the proposed method. Three of the target PCBs were detected in one lettuce sample with the total concentration of 17.9 *μ*g/kg.

## 1. Introduction

Polychlorinated biphenyls (PCBs), a group of 209 congeners that differ in the number and position of chlorine atoms on the biphenyl ring, are anthropogenic chemicals that have been classified and regulated as one of the 12 persistent organic pollutants under the Stockholm Convention on POPs [1]. PCBs have physicochemical properties that are industrially useful, including nonflammability, chemical stability, high boiling point, and electrical insulating properties, which has led to their mass production and widespread use in electrical transformers, capacitors, regulators, hydraulic fluids, plasticizers, and lubricants [2]. Despite the limited production or the ban of PCBs since the late 1970s due to their toxic, bioaccumulative, and carcinogenic behavior, some industrial equipment containing PCBs has remained in operation [3, 4]. Large amounts of PCBs have been released into the environment, and congeners are still frequently detected in environmental media including soil [5], sediment [6], water [7], and air [8]. Given their extensive historical usage, environmental persistence, and nondegradable nature, PCBs are found in a variety of foodstuffs originating from contaminated soils worldwide [9, 10]. Dietary intake, for example, through vegetable consumption, has been considered as a major pathway of human exposure to PCBs [11]. Vegetables, e.g., cabbage, mustard, lettuce, and kale, collected from a production site near an electroplating factory in China were found to contain 13.5–30.5 *μ*g/kg of PCBs [12]. An average PCB concentration of up to 228.68 *μ*g/kg was found in vegetables grown near an electronic waste dismantling area on the southeast coast of China [13]. Because of their lipophilic nature, PCBs accumulate readily in the human body via the food chain, which may negatively impact human health, including tumor promotion and teratogenicity, and perturbation of the immune, endocrine, and nervous systems [14–16]. Hence, monitoring PCB contamination in food is of critical importance to protect human health and safety.

Recently, a variety of analytical methods have been developed for the measurement of PCBs, including gas chromatography-electron capture detector (GC-ECD) [8], gas chromatography-mass spectrometry (GC-MS) [5, 9, 10, 13, 17], and gas chromatography-tandem mass spectrometry (GC-MS/MS) [6, 12, 18]. However, these methods were typically applied to environmental samples, such as soil [19, 20] and sediment [21, 22]. Very few methods have been reported for measuring PCB levels in vegetable samples [10, 12], and the procedures for sample pretreatment are usually based on the Soxhlet extraction [4, 17], liquid-liquid extraction [9], and/or microwave-assisted extraction [19] techniques, coupled with cleanup methods involving column chromatography [6, 10, 18], solid-phase extraction [13], and concentrated sulfuric acid [20]. Most of these approaches are cumbersome, time-consuming, and expensive and often consume large volumes of toxic solvents. In response to these limitations, dispersive solid-phase extraction (d-SPE), emerging as an alternative to conventional SPE procedure, has attracted much attention due to simple operation, low solvent consumption, safety, and high efficiency [23]. In this method, the sorbent is directly added to the sample extract, and the resulting mixture is shaken to achieve rapid purification through the interaction between the sorbents and the interfering substances in the sample matrices [24]. In this case, the sorbent is a key factor that determines the selectivity and sensitivity. Multiwalled carbon nanotubes (MWCNTs) are nanosized hollow tubes formed from curved graphite-like planes composed of six-membered rings of carbon [25], which are characterized by desirable physicochemical properties including larger specific surface area, higher adsorption capacity, and stronger adsorption capability than primary secondary amine (PSA) and octadecylsilane (C18). As new high-performance sorbents, MWCNTs have been widely applied to determine different compounds in various food samples [26–31]. However, MWCNTs have been treated in mixed acid reagents to form a high density of carboxyl groups on their surface, shortening the carbon nanotubes as well as removed impurities through the acidification treatment, which significantly increased their dispersion in water. Carboxylated multiwalled carbon nanotubes (MWCNTs-COOH) have higher specific surface area and better dispersion characteristics than MWCNTs (Supplementary Material, [Supplementary-material supplementary-material-1]). Previous work has shown that MWCNTs-COOH exhibited a better cleanup performance as d-SPE sorbent in the analysis of pesticides in vegetables [32] and PCBs in fresh tea leaves or made teas [33, 34].

In this study, we aimed to establish a simple, rapid, and reliable method based on d-SPE with MWCNTs-COOH as sorbent for the determination of PCBs in vegetables. To evaluate the proposed method, 18 PCBs in four vegetable matrices (cucumber, tomato, lettuce, and cabbage) were analyzed and GC-MS was used to identify each analyte and determine its concentration in each sample matrix. After validation, this method was utilized for the analysis of the target analytes in real vegetable samples.

## 2. Materials and Methods

### 2.1. Chemicals and Reagents

The compounds in Table 1 were provided in a standard solution from o2si smart solutions (Charleston, SC, USA) containing 18 PCB congeners (indicator PCBs: 28, 52, 101, 118, 138, 153, and 180; dioxin-like PCBs: 77, 81, 105, 114, 118, 123, 126, 156, 157, 167, 169, and 189) at 10 mg/L each in *n*-hexane. MWCNTs-COOH (length, 10–30 *μ*m; diameter, 10–20 nm) were purchased from Nanjing XFNANO Materials Tech Co., Ltd. (Nanjing, China). Primary secondary amine (PSA; particle size, 40–60 *μ*m), graphitized carbon black (GCB; particle size, 40–120 *μ*m), and octadecylsilane (C18; particle size, 40–60 *μ*m) were from Tianjin Bonna-Agela Technologies Co., Ltd. (Tianjin, China). *n*-Hexane was of HPLC grade and purchased from Oceanpak Alexative Chemical., Ltd (Goteborg, Sweden). All other chemical reagents like acetonitrile, acetone, toluene, anhydrous Na_2_SO_4_, and anhydrous MgSO_4_ in this research were of analytical grade or better and purchased from Shanghai Sinopharm Chemical Reagent Co., Ltd. (Shanghai, China).

### 2.2. Apparatus and GC-MS Conditions

A KQ-500DE digital ultrasonic cleaner was purchased from Kunshan Ultrasonic Instruments Co., Ltd. (Kunshan, China). A TG16-WS high-speed centrifuge was purchased from Xiangyi Instrument (Hunan, China). An HSC-24B nitrogen concentrator was purchased from Tianjin Heng Ao Technology Development Co., Ltd. (Tianjin, China).

Analysis of the 18 PCBs was conducted on a 7890B gas chromatograph (Agilent, USA) with a 5977A mass spectrometric detector (Agilent, USA). An Agilent HP-5MS analytical column (30 m × 0.25 mm i.d. × 0.25 *μ*m film thickness) was used for GC separation with helium (99.9999%) as the carrier gas at a constant flow rate of 1.2 mL/min. The GC oven temperature was initially 80°C and increased to 180°C at 20°C/min. The oven temperature was then increased to 230°C at 3°C/min and finally to 280°C at 10°C/min. Each temperature setting was held for 2 min. The inlet temperature was 250°C, and 1 *μ*L of the extract was injected into the gas chromatograph in the splitless mode.

The mass spectrometer employed an electron impact (EI) ionizer at 70 eV and was operated in the selective ion monitoring (SIM) mode. The temperature of the quadrupole was set at 150°C. The temperatures of the ion source and the mass spectrometer transfer line were both set at 280°C. Analyses were performed with a solvent delay of 5 min. One quantitative ion and at least two qualitative ions were selected for each PCB.

### 2.3. Preparation of Standard Solutions

One milliliter of PCB stock standard solution (10 mg/L) was transferred to a 10-milliliter volumetric flask and diluted to the mark with *n*-hexane to obtain a standard solution containing 1 mg/L of each of the 18 PCBs. The newly prepared standard solution was stored in a refrigerator at 4°C. Before use, the standard solution was removed from the refrigerator and allowed to reach room temperature before further dilution with *n*-hexane to obtain working standard solutions with concentrations of 5, 10, 50, 100, and 500 *μ*g/L.

The blank sample was extracted by the proposed method, and then the extract was evaporated to dryness under a gentle stream of nitrogen. The residues for the 1 mL blank matrix extract were redissolved in 1 mL of the same concentration of working standard solution to realize matrix-matched standard solutions of 5, 10, 50, 100, and 500 *μ*g/kg.

All solutions were stored in the refrigerator at 4°C before analysis.

### 2.4. Sample Preparation

Vegetable samples, including cucumber, tomato, lettuce, and cabbage, were collected from a local vegetable farm in Suzhou in eastern China. They were homogenized with a blender and kept at −20°C before analysis. Blank samples were used for validation studies and to prepare matrix-matched standard mixtures. Samples to be used for recovery studies were spiked with a known amount of the PCB standard solution and left for 1 h before beginning the extraction.

To prepare vegetable extracts, 5.0-gram aliquots of homogenized vegetables were weighed into 50-milliliter centrifuge tubes. Each tube was then charged with 10 mL of acetone : *n*-hexane (1 : 2, v/v) and 2 g of anhydrous Na_2_SO_4_. The tubes were vortexed for 1 min, followed by ultrasonic-assisted extraction (UAE) for 15 min at 500 W and 40 kHz. Following extraction, the centrifuge tubes were centrifuged for 4 min at 8,000 rpm. Two milliliters of the supernatant was transferred to a new, clean 10-milliliter centrifuge tube and reduced to near dryness by evaporating with a gentle stream of nitrogen gas at 80°C. The extract was redissolved in 5 mL of toluene for future cleanup.

For cleanup, the above extract was placed in 4-milliliter microcentrifuge tubes containing 0.02 g of MWCNTs-COOH and 0.1 g of anhydrous MgSO_4_. The mixture was shaken vigorously for 2 min and centrifuged for 5 min at 9,000 rpm. The supernatant was removed and evaporated to dryness at 80°C. The resulting residue was redissolved in 1 mL of n-hexane and filtered through a 0.22-micrometer filter membrane prior to GC-MS analysis.

### 2.5. Method Validation

Four representative matrices were selected for validation purposes: cucumber (cucurbitaceous vegetable; high water and chlorophyll content), tomato (solanaceous vegetable; high water, sugar, and lipid content and low or no chlorophyll content), cabbage and lettuce (leafy vegetable; high water, protein, and chlorophyll content) [35]. The following parameters were determined during validation of the analytical method: linearity, matrix effect, accuracy and precision, limit of detection (LOD), and limit of quantification (LOQ). To avoid the matrix effect, matrix-matched calibration of four vegetable samples was carried out to evaluate linearity. Accuracy and precision were determined by the recovery and repeatability of six replicate analyses for each sample matrix at three spiked concentrations (5, 10, and 100 *μ*g/kg). The LOD and LOQ were defined as the concentration of the analyte giving a signal-to-noise (S/N) ratio of 3 and 10, respectively, for a given target ion. Both LOD and LOQ were estimated by analyzing spiked samples containing the lowest concentration of the analyte. According to European Union (EU) guidelines SANTE/11813/2017, recoveries were considered satisfactory in the range of 70–120% with an associated RSD less than or equal to 20% [36].

## 3. Results and Discussion

### 3.1. Development of GC-MS Method

PCBs are a class of nonpolar compounds containing benzene rings. In order to obtain the best separation conditions for the analytes, a low-polarity and low-bleed HP-5MS capillary column was selected for chromatographic separation with the optimal parameters including chromatographic temperature program, carrier gas flow rate, inlet temperature according to the sensitivity, and analytic time of the target analytes. Under the optimal GC conditions described in Section 2.2, the total ion chromatogram (TIC) was obtained by analyzing 500 *μ*g/L of the working standard solution in the range of *m/z* 50–450 by GC-MS in the full-scan mode. Based on the TIC spectrum, the retention time of each analyte was identified by searching in the NIST 2011 database. In the mass spectrum after subtracting the background, the characteristic ions with high abundance, large *m/z*, and less matrix interference were selected. To obtain a maximum signal for each analyte, enough dwell time and adequate acquisition points were needed for each chromatographic peak. In the GC-MS acquisition method, the analytes were divided into groups, as many as possible according to their retention times. One quantitative and two qualitative ions were monitored for each analyte by GC-MS.

**Figure 1:**
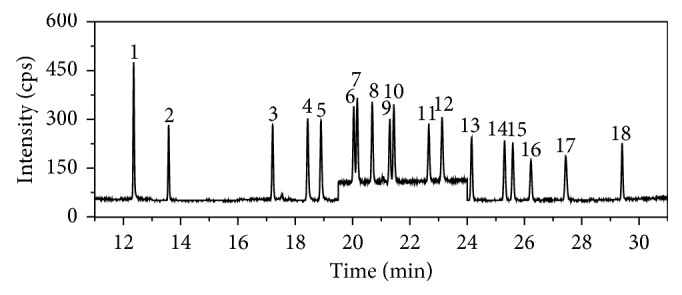
SIM chromatogram of 18 PCB (10 *μ*g/L) standard solutions: peaks 1–18 are PCB28, PCB52, PCB101, PCB118, PCB153, PCB138, PCB180, PCB81, PCB77, PCB123, PCB114, PCB105, PCB126, PCB167, PCB156, PCB157, PCB169, and PCB189.

The MS features used to identify and quantitate each of the 18 PCBs are shown in Table 1. A GC-MS chromatogram obtained for each PCB at 10 *μ*g/L is shown in Figure 1.

### 3.2. Selection of Extraction Conditions

#### 3.2.1. Extraction Solvent

**Figure 2:**
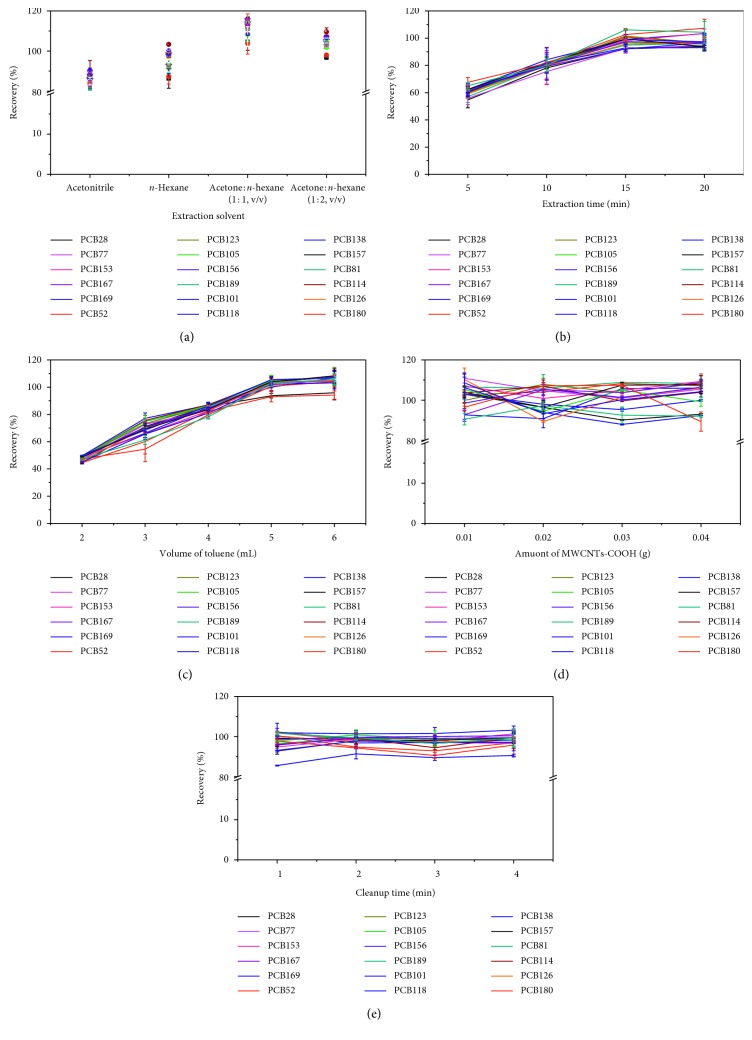
Optimization of major factors affecting extraction and cleanup efficiency (*n*=3): (a) extraction solvent; (b) extraction time; (c) volume of toluene; (d) amount of MWCNTs-COOH; (e) cleanup time.

The type of extraction solvent is vital for the extraction efficiency. Therefore, extraction solvents should be carefully taken into account. In the proposed method, several extraction solvents were examined, including *n*-hexane, mixture of acetone : *n*-hexane (1 : 1 and 1 : 2, by volume), and acetonitrile which are most frequently applied in the analysis of the target compounds in solid matrices. The efficiency was evaluated as recoveries obtained by spiking the blank cucumber sample with 18 PCBs at 10 *μ*g/kg. As shown in Figure 2(a), the recoveries of different extraction solvents ranked in the following order: acetone : *n*-hexane(1 : 1, v/v)>acetone : *n*-hexane(1 : 2, v/v)>*n*-hexane>acetonitrile. Although acetone : *n*-hexane (1 : 1, v/v) had exhibited the highest extraction efficiency among the solvents used, the color density of the extract was greater with this solvent than with other tested solvents, indicating more coelution of organic matters. Thus, acetone : *n*-hexane (1 : 2, v/v) was adopted as the optimum extraction solvent since it provided better recoveries for the target analytes and less sample matrix residues were found.

The volume of the extraction solvent is also an important factor affecting the extraction efficiency. To examine the effect of the volume of the solvent on extraction efficiency, the volume of acetone : *n*-hexane (1 : 2, v/v) was varied over the range of 5–15 mL. The results (data not shown) showed that extraction efficiency was greatest when using 10 mL of 1 : 2 acetone : *n*-hexane, yielding recovery rates of 99.4%–110.5%. Therefore, 10 mL was selected as the optimum extraction volume in the following experiments.

#### 3.2.2. Extraction Time

Ultrasonic-assisted extraction (UAE) is an effective means of extracting target compounds from complex matrices. UAE boasts several advantages over conventional extraction methods, including lower solvent volumes, faster extraction, and increased recovery. Extraction time is usually a very important parameter in most extraction procedures since it influences the partition of the target analytes between the sample solution and the organic phase. In this study, to extract the maximum amount of analytes, the effect of extraction time on the extraction efficiency was examined from 5 to 20 min. As shown in Figure 2(b), the extraction recoveries obtained by spiking the blank cucumber sample with 18 PCBs at 10 *μ*g/kg increased as the extraction time increased. However, extraction times longer than 15 min did not produce a significant enhancement of the extraction efficiency. This indicated that the kinetics of UAE for the analytes was quite fast and the extraction equilibrium could be reached within 15 min as well as provided high extraction recoveries for the analytes. Therefore, 15 min was selected as the optimum sonication time.

### 3.3. Optimization of Sample Cleanup

Cleanly extracting target analytes from vegetable samples can be difficult. Vegetables contain high concentrations of plant pigments that are often coextracted with analytes, resulting in large matrix effects and instrument contamination. Therefore, extracts from such samples need to be cleaned prior to analysis. Here, matrix impurities were removed from sample extracts by the d-SPE method. The effects of cleanup duration and the type and amount of sorbent were investigated in terms of cleanup efficiency.

#### 3.3.1. Type and Amount of Sorbent

**Figure 3:**
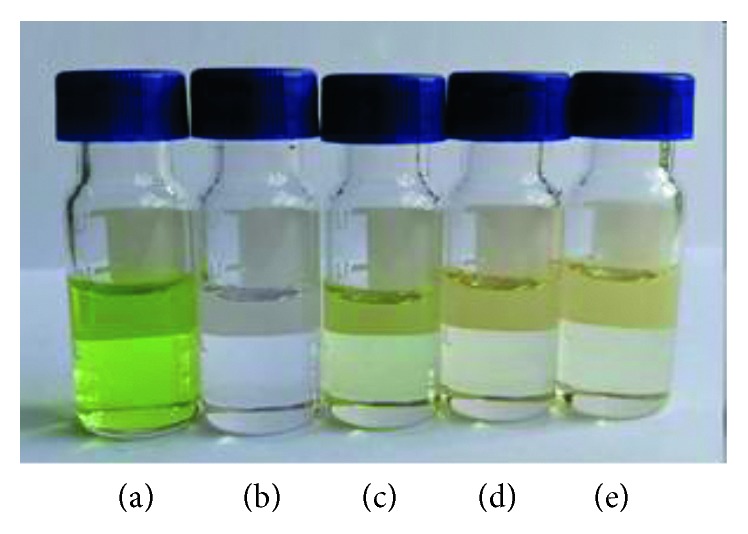
Photography of cleanup efficiency using different d-SPE absorbents: (a), without cleanup; (b), MWCNTs-COOH; (c), C18; (d), PSA; (e), GCB.

**Figure 4:**
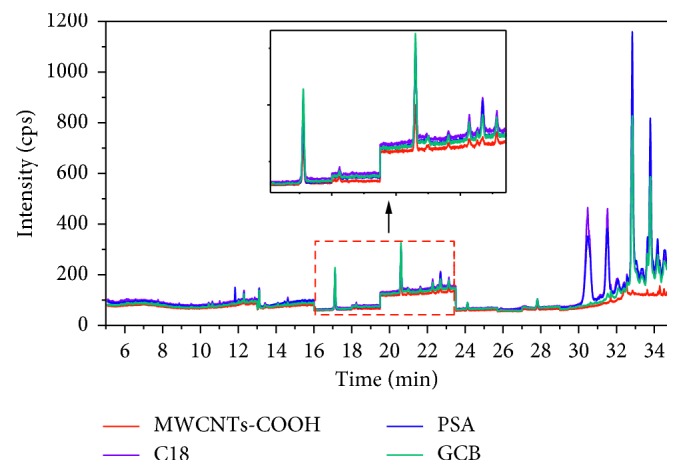
Chromatogram of the blank cucumber extract prepared using different adsorbents during the d-SPE procedure.

The efficiency of d-SPE depends on the type and quantity of sorbent. A suitable sorbent would remove major matrix impurities without retaining any target analyte. In the present work, the comparison for the cleanup effect of the cucumber sample by using MWCNTs-COOH, GCB, C18, or PSA at the amount of 0.02 g was evaluated. As shown in Figure 3, the final cucumber sample without cleanup was green and turbid, and the sample purified by GCB, C18, or PSA retained some of their initial color. All in all, the cleanup effect of MWCNTs-COOH was the best which was almost colorless and clear.  Figure 4 shows the GC-MS-SIM chromatogram of the blank cucumber extract with different adsorbents' cleanup. It can be seen that many of the interference substances are removed by the MWCNTs-COOH after the d-SPE cleanup procedure, which enhances the cleanup performance. After cleanup with MWCNTs-COOH, less interference appeared in the chromatogram of the blank cucumber sample, and the baseline became lower than GCB, C18, or PSA cleanup. An explanation to this observation could be that due to their special physical structure, MWCNTs-COOH have large surface area, which gives them excellent adsorption ability for a wide range of substances; thus, the interferences from the extract could be absorbed on the surface of the nanotubes. On the contrary, nanotubes have hollow cylindrical structures, and some interfering compounds with small chemical structures could thread the carbon layer into the cylinder. When the absorption reaches saturation, the nanotubes would not absorb these interfering substances any more. Therefore, the mechanism of matrix interference removal by MWCNTs-COOH is probably based on two types of interactions between the MWCNTs-COOH and interference substances, including adsorption on the surface of MWCNTs-COOH, and the absorptive action of nanotubes [28].

Moreover, the cleanup efficiency was also estimated by the recovery assays spiking the blank cucumber sample at 10 *μ*g/kg employing aforementioned sorbents at the amount of 0.02 g. The results showed that recovery rates obtained with C18 and PSA as d-SPE sorbents were 80.2%–113.1% and 74.9%–105.3%, respectively, while most of those with MWCNTs-COOH and GCB as d-SPE sorbents were less than 70%. Moreover, the recovery rates of four PCBs, including PCB81, PCB77, PCB126, and PCB169, were 0. This might be due to the strong affinity and *π*−*π* bonding between planar PCBs and MWCNTs-COOH or GCB [37, 38]. This indicates that the proposed method may not be suitable for determination of compounds containing planar polycyclic structures. To overcome this problem, the cleanup procedure was conducted after replacing the extract solvent with toluene. The benzene group from toluene may compete with MWCNTs-COOH or GCB and reduce the tendency of PCBs being absorbed. This dramatically increased recovery rates of the PCBs from 0%–81.6% to 91.8%–100.2% and 0%–89.7% to 88.8%–99.3% for MWCNTs-COOH and GCB, respectively. The volume of toluene (2, 3, 4, 5, or 6 mL) used to redissolve the extract was then evaluated in terms of analyte recovery from cucumber matrices containing PCBs (10 *μ*g/kg). As shown in Figure 2(c), 5 mL of toluene was sufficient to obtain recovery rates of 93.0%–105.5%. Further increases in the volume of toluene did not yield a significant increase in recovery. Therefore, 5 mL was selected as the optimum amount of toluene for redissolving dried sample extracts prior to the d-SPE cleanup. Based on the acceptable recovery rates and good cleanup performances, MWCNTs-COOH were used as the d-SPE sorbent in the further experiments. The amount of sorbent is an important factor influencing cleanup performance and analyte recovery. Insufficient sorbent yields poor purification. Excess sorbent, while yielding high-purity product, can also significantly lower recovery rates. The effects of the amount of MWCNTs-COOH in the cleanup mixture (0.01, 0.02, 0.03, or 0.04 g) on the recovery rates of PCBs spiked in cucumber matrices were assessed. The data in Figure 2(d) show that as the amount of MWCNTs-COOH in the cleanup mixture increased, the sample extracts gradually became lighter in color and eventually transparent, while recovery rates dropped. With 0.01 g and 0.02 g of MWCNTs-COOH, recovery rates in cucumber matrices were 90.5%–110.8% and 89.3%–107.9%, respectively. However, with 0.02 g of MWCNTs-COOH, the cleaned extract was visibly lighter in color and more transparent. Thus, 0.02 g of MWCNTs-COOH was selected as the optimal amount for the d-SPE procedure.

#### 3.3.2. Cleanup Time

Cleanup time, i.e., the amount of time that the d-SPE sorbent is mixed with the extract, can also affect recovery rates and cleanup efficiency. The blank cucumber sample spiked with 10 *μ*g/kg of 18 PCBs was vortexed for 1, 2, 3, or 4 min, and the results are shown in Figure 2(e). As it can be seen, with a cleanup time of 2 min, the recovery rates were in the range of 91.3%–101.4%, and prolonged cleanup times resulted in lower recovery. Therefore, 2 min was idnetified as the optimum vortex duration during the d-SPE cleanup process.

On the basis of the observations mentioned above, the optimum UAE-MWCNTs-d-SPE conditions were determined as follows: type of extraction solvent, acetone : *n*-hexane (1 : 2, v/v); volume of extraction solvent, 10 mL; extraction time, 15 min; volume of toluene, 5 mL; amount of MWNCTs-COOH, 0.02 g; and cleanup time, 2 min.

### 3.4. Method Validation

Validation of the developed method in terms of matrix effect, linearity, accuracy and precision, LODs, and LOQs was performed according to EU guidelines SANTE/11813/2017 [36]. The results were summarized in Supplementary Material [Supplementary-material supplementary-material-1] and [Supplementary-material supplementary-material-1].

#### 3.4.1. Matrix Effect

The occurrence of matrix effect (ME) is regarded as a signal suppression or enhancement of the analyte due to the coelution of matrix components, which can interfere significantly with the analysis of the target analytes and affect the accuracy and precision of the method [39]. To evaluate the impact of the matrix effect on the analytes, the slopes obtained in the calibration curves with matrix-matched standards were compared with those obtained with solvent-based standards, calculating matrix/solvent slope ratios for each of the 18 PCBs in the four different matrices. An ME value larger than 1 may represent the matrix enhancement effect, and a matrix suppression effect may resulted in an ME value smaller than 1. In this work, the matrix effect was relatively weak and could be ignored when the ME value was in the range of 0.9–1.1; if the ME value was larger than 1.10 and smaller than 0.90, the matrix enhancement effect and matrix suppression effect were considered [27]. As shown in Supplementary Material [Supplementary-material supplementary-material-1], there was a matrix enhancement effect for all the tested compounds. Therefore, quantification was performed by using the matrix-matched standards throughout the developed method to minimize errors due to matrix effect.

#### 3.4.2. Linearity

Linearity was evaluated across a concentration range of 5–500 *μ*g/kg with five calibration standards (5, 10, 50, 100, and 500 *μ*g/kg) for all PCBs in cucumber, tomato, lettuce, and cabbage matrices. The data in Supplementary Material [Supplementary-material supplementary-material-1] show good linearity for all PCBs with calibration curve coefficients (*r*) greater than 0.9993.

#### 3.4.3. LODs and LOQs

The LODs and LOQs were defined by the signal-to-noise (S/N) ratio of 3 and 10 from the lowest concentration levels of the spiked samples. As demonstrated in Supplementary Material [Supplementary-material supplementary-material-1], the LODs and LOQs for all PCBs in cucumber, tomato, lettuce, and cabbage matrices ranged from 0.3 to 1.4 and from 0.8 to 4.5 *μ*g/kg, respectively.

#### 3.4.4. Accuracy and Precision

To test the accuracy and precision of the developed method, recovery assays were performed for each matrix at three spiked levels (5, 10, and 100 *μ*g/kg) with six replicates. The accuracy was estimated by recoveries (%), and the precision was evaluated by RSDs (%) of the spiked samples. As indicated in Supplementary Material [Supplementary-material supplementary-material-1], the recoveries of all analytes were in the range of 84.5%–116.5% (93.8%–115.2% for cucumber, 84.5%–107.8% for tomato, 85.0%–113.9% for lettuce, and 88.5%–116.5% for cabbage) with RSDs (*n*=6) lower than 17.6%, which were within acceptable range [36]. Satisfactory recoveries were obtained for the samples assayed, which were mainly attributed to the solubility of PCBs in the extraction solvent and the stability of PCBs limiting their losses during extraction.

### 3.5. Method Application

To demonstrate the applicability of the method, it was applied to the analysis of twelve real vegetable samples including three cucumber samples, three tomato samples, three lettuce samples, and three cabbage samples collected from vegetable farms in Suzhou, China. These samples were prepared following the above procedure. After GC-MS analysis, three of target PCBs (PCB118, PCB153, and PCB138) were detected in one lettuce sample at concentrations of 5.1, 6.6, and 6.2 *μ*g/kg, respectively. No other target PCBs were detected in these samples.

### 3.6. Comparison with Other Reported Methods

A comparative study of our developed method to other reported sample preparation procedures for determination of PCBs in vegetable samples was performed, and the results are presented in Table 2. It can be seen that the developed method was cost-effective, convenient and rapid with less consumption of organic solvent, and shorter preparation time than previous reports.

## 4. Conclusion

In this study, a simple, reliable, and rapid method for analysis of 18 PCBs in vegetable samples has been developed based on MWCNTs-COOH cleanup combined with GC–MS determination, which provides a powerful tool for measuring PCB levels in vegetable samples. The sample preparation procedure is straightforward and easy to perform, and the cleanup only needs 0.02 g of MWCNTs-COOH by the mode of d-SPE. The matrix effects for the targeted PCBs were evaluated based on the slopes of calibration curves. The calibration parameters of the method including linearity, accuracy, precision, LOD, and LOQ were examined, which showed MWCNTs-COOH could be used as a promising sorbent in the analysis of PCBs. Compared with other reported methods, when the developed method was applied, the whole pretreatment time was shortened, and the consumption of toxic organic solvents was reduced significantly.

## Figures and Tables

**Table 1:** Retention times, characteristic ions, and their relative abundance of the 18 PCBs analyzed in the present study.

No	IUPAC number	IUPAC name	Formula	Retention time (min)	Time window (min)	Characteristic ions (*m/z*)	Relative abundances (%)
1	PCB28	2,4,4′-trichlorobiphenyl	C_12_H_7_Cl_3_	12.373	5.00–13.00	256^*∗*^/186/150	100/68/18
2	PCB52	2,2′,5,5′-tetrachlorobiphenyl	C_12_H_6_Cl_4_	13.597	13.00–16.00	292^*∗*^/220/110	100/82/20
3	PCB101	2,2′,4,5,5′-pentachlorobiphenyl	C_12_H_5_Cl_5_	17.233	16.00–18.00	326^*∗*^/254/127	100/59/18
4	PCB81	3,4,4′,5-tetrachlorobiphenyl	C_12_H_6_Cl_4_	18.448	18.00–19.50	292^*∗*^/220/110	100/40/15
5	PCB77	3,3′,4,4′-tetrachlorobiphenyl	C_12_H_6_Cl_4_	18.913		292^*∗*^/220/110	100/43/13
6	PCB123	2′,3,4,4′,5-pentachlorobiphenyl	C_12_H_5_Cl_5_	20.068	19.50–22.00	326^*∗*^/254/127	100/40/11
7	PCB118	2,3′,4,4′,5-pentachlorobiphenyl	C_12_H_5_Cl_5_	20.184		326^*∗*^/254/127	100/35/10
8	PCB114	2,3,4,4′,5-pentachlorobiphenyl	C_12_H_5_Cl_5_	20.710		326^*∗*^/254/127	100/46/12
9	PCB153	2,2′,4,4′,5,5′-hexachlorobiphenyl	C_12_H_4_Cl_6_	21.329		360^*∗*^/290/145	100/65/24
10	PCB105	2,3,3′,4,4′-pentachlorobiphenyl	C_12_H_5_Cl_5_	21.469		326^*∗*^/254/127	100/36/14
11	PCB138	2,2′,3,4,4′,5′-hexachlorobiphenyl	C_12_H_4_Cl_6_	22.689	22.00–24.00	360^*∗*^/290/145	100/62/20
12	PCB126	3,3′,3,4,4′,5-pentachlorobiphenyl	C_12_H_5_Cl_5_	23.136		326^*∗*^/254/127	100/30/13
13	PCB167	2,3′,4,4′,5,5′-hexachlorobiphenyl	C_12_H_4_Cl_6_	24.174	24.00–25.00	360^*∗*^/290/145	100/38/16
14	PCB156	2,3,3′,4,4′,5-hexachlorobiphenyl	C_12_H_4_Cl_6_	25.328	25.00–26.00	360^*∗*^/290/145	100/34/15
15	PCB157	2,3,3′,4,4′,5′-hexachlorobiphenyl	C_12_H_4_Cl_6_	25.622		360^*∗*^/290/145	100/45/17
16	PCB180	2,2′,3,4,4′,5,5′-heptachlorobiphenyl	C_12_H_3_Cl_7_	26.250	26.00–27.00	394^*∗*^/324/162	100/72/36
17	PCB169	3,3′,4,4′,5,5′-hexachlorobiphenyl	C_12_H_4_Cl_6_	27.470	27.00–29.00	360^*∗*^/290/145	100/40/17
18	PCB189	2,3,3′,4,4′,5,5′-hexachlorobiphenyl	C_12_H_3_Cl_7_	29.425	29.00–31.00	394^*∗*^/324/162	100/50/23

^*∗*^Quantitative ion.

**Table 2:** Comparison of the proposed method with other methods reported in the literature for the determination of PCBs in vegetable samples.

Extraction	Cleanup	Detection	Volume of extraction solvent (mL)	Sample preparation time (h)	LOD (*μ*g/kg)	Recovery	Reference
Soxhlet extraction	Concentrated sulfuric acid and silica gel/alumina column	GC-MS	—	>72	0.01	92.5 ± 8.5	[10]
UAE	C18 SPE	GC-MS	70	>12	1.43	80–87	[13]
UAE	Sulfuric acid-silica gel SPE	GC-ECD	120	>2.2	0.01–0.015	87	[40]
Mechanical shaking	Concentrated sulfuric acid	GC-ECD	≥150	>25.5	0.14–0.16 pg	75–85	[41]
Soxhlet extraction	Concentrated sulfuric acid and Florisil SPE	GC-MS	100	>22	0.04–0.43	87.5–106.8	[42]
UAE	MWCNTs-COOH d-SPE	GC-MS	10	<1.2	0.3–1.4	84.5–116.5	This work

## Data Availability

The data used to support the findings of this study are included within the article and the supplementary information file.

## Supplementary Materials

Supplementary Materials (1) Table S1 shows a comparison between MWCNTs and MWCNTs-COOH. (2) Table S2 shows linear regression equations, correlation coefficients, linear ranges, ME, LODs, and LOQs for the 18 PCBs of the proposed method. (3) Table S3 shows spiked recoveries and RSDs of the 18 PCBs in the tested vegetable matrices of the proposed method.
