# Supplementary material for: Carboxylated Multiwalled Carbon Nanotubes as Dispersive Solid-Phase Extraction Sorbent to Determine Eighteen Polychlorinated Biphenyls in Vegetable Samples by Gas Chromatography-Mass Spectrometry
Source: J Anal Methods Chem. 2019 Aug 20;2019:4264738. doi: 10.1155/2019/4264738 (PMC6720675; doi:10.1155/2019/4264738)
Supplement: Supplementary Materials — (1) Table S1 shows a comparison between MWCNTs and MWCNTs-COOH. (2) Table S2 shows linear regression equations, correlation coefficients, linear ranges, ME, LODs, and LOQs for the 18 PCBs of the proposed method. (3) Table S3 shows spiked recoveries and RSDs of the 18 PCBs in the tested vegetable matrices of the proposed method. [file 4264738.f1.doc]

**Supplementary Material**

T_ABLE_ S1: A comparison between MWCNTs and MWCNTs-COOH

| CNTs | Length  (μm) | OD  (nm) | Purity  (%) | Making method | SAA  (m^2^/g) | -COOH Content  (wt%) |
| --- | --- | --- | --- | --- | --- | --- |
| MWCNTs | 10-30 | 10-20 | >95% | CVD | >120 | 0 |
| MWCNTs-COOH | 10-30 | 10-20 | >95% | CVD | >150 | 2.00 |

**OD**: Outer Diameter; **SAA**: Special Surface Area; **CVD**: Chemical Vapor Deposition; Data are obtained from Nanjing XFNANO Materials Tech Co., Ltd. and the website is https://en.xfnano.com/

T_ABLE_ S2: Linear regression equations, correlation coefficients, linear ranges, ME, LODs and LOQs of the 18 PCBs

| Analyte | Matrix | Regression equation | *r* | Linear range  (μg/kg) | ME | LOD  (μg/kg) | LOQ  (μg/kg) |
| --- | --- | --- | --- | --- | --- | --- | --- |
| PCB28 | Hexane | *y*=173043*x*-10.078 | 0.9999 | 5-500 | - | - | - |
|  | Cucumber | *y*=194715*x*-316.33 | 1.0000 | 5-500 | 1.13 | 0.3 | 0.8 |
|  | Tomato | *y*=181977*x*+214.52 | 0.9999 | 5-500 | 1.05 | 0.4 | 1.2 |
|  | Lettuce | *y*=190305*x*+245.30 | 0.9999 | 5-500 | 1.10 | 0.4 | 1.3 |
|  | Cabbage | *y*=216020*x*-1318.7 | 0.9997 | 5-500 | 1.25 | 0.4 | 1.2 |
| PCB52 | Hexane | *y=*89322*x+*41.223 | 0.9999 | 5-500 | - | - | - |
|  | Cucumber | *y*=111483*x*-436.31 | 0.9999 | 5-500 | 1.25 | 0.5 | 1.6 |
|  | Tomato | *y*=97089*x*+46.996 | 1.0000 | 5-500 | 1.09 | 0.7 | 2.1 |
|  | Lettuce | *y*=98235*x*+123.91 | 0.9999 | 5-500 | 1.10 | 0.9 | 2.7 |
|  | Cabbage | *y*=110901*x*-645.08 | 0.9997 | 5-500 | 1.24 | 0.7 | 2.3 |
| PCB101 | Hexane | *y=*108202*x+*41.338 | 0.9999 | 5-500 | - | - | - |
|  | Cucumber | *y*=129570*x*-328.30 | 0.9999 | 5-500 | 1.20 | 0.5 | 1.6 |
|  | Tomato | *y*=117875*x*+115.66 | 0.9999 | 5-500 | 1.09 | 0.7 | 2.1 |
|  | Lettuce | *y*=121392*X* +137.00 | 0.9999 | 5-500 | 1.12 | 0.8 | 2.6 |
|  | Cabbage | *y*=137084*x*-772.07 | 0.9996 | 5-500 | 1.27 | 0.7 | 2.3 |
| PCB81 | Hexane | *y=*149277*x-*3.9724 | 0.9999 | 5-500 | - | - | - |
|  | Cucumber | *y*=172471*x*-389.53 | 1.0000 | 5-500 | 1.16 | 0.4 | 1.3 |
|  | Tomato | *y*=161429*x*+170.94 | 0.9999 | 5-500 | 1.08 | 0.5 | 1.7 |
|  | Lettuce | *y*=169728*x*+231.72 | 0.9999 | 5-500 | 1.13 | 0.7 | 2.1 |
|  | Cabbage | *y*=191102*x*-1201.4 | 0.9996 | 5-500 | 1.28 | 0.6 | 1.8 |
| PCB77 | Hexane | *y=*151256*x-*4.7629 | 0.9999 | 5-500 | - | - | - |
|  | Cucumber | *y*=173140*x*-368.51 | 1.0000 | 5-500 | 1.14 | 0.4 | 1.3 |
|  | Tomato | *y*=163030*x*+195.09 | 0.9999 | 5-500 | 1.08 | 0.6 | 1.7 |
|  | Lettuce | *y*=170643*x*+277.77 | 0.9999 | 5-500 | 1.13 | 0.7 | 2.1 |
|  | Cabbage | *y*=192606*x*-1216.6 | 0.9996 | 5-500 | 1.27 | 0.6 | 1.9 |
| PCB123 | Hexane | *y=*129076*X -*48.424 | 0.9999 | 5-500 | - | - | - |
|  | Cucumber | *y*=151238*x*-399.14 | 0.9999 | 5-500 | 1.17 | 0.5 | 1.4 |
|  | Tomato | *y*=140083*x*+126.82 | 0.9999 | 5-500 | 1.09 | 0.6 | 1.9 |
|  | Lettuce | *y*=145790*x*+301.65 | 0.9999 | 5-500 | 1.12 | 0.7 | 2.2 |
|  | Cabbage | *y*=164758*x*-991.67 | 0.9995 | 5-500 | 1.28 | 0.7 | 2.1 |
| PCB118 | Hexane | *y=*144551*x-*11.242 | 0.9999 | 5-500 | - | - | - |
|  | Cucumber | *y*=165489*x*-410.80 | 0.9999 | 5-500 | 1.14 | 0.4 | 1.3 |
|  | Tomato | *y*=153886*x*+219.45 | 0.9999 | 5-500 | 1.06 | 0.6 | 1.8 |
|  | Lettuce | *y*=161255*x*+209.85 | 0.9999 | 5-500 | 1.13 | 0.7 | 2.1 |
|  | Cabbage | *y*=182471*x*-1058.1 | 0.9996 | 5-500 | 1.26 | 0.6 | 1.8 |
| PCB114 | Hexane | *y=*134913*x-*40.182 | 0.9999 | 5-500 | - | - | - |
|  | Cucumber | *y*=156654*x*-394.87 | 0.9999 | 5-500 | 1.16 | 0.4 | 1.4 |
|  | Tomato | *y*=145124*x*+179.35 | 0.9999 | 5-500 | 1.08 | 0.6 | 1.9 |
|  | Lettuce | *y*=152009*x*+160.35 | 0.9999 | 5-500 | 1.12 | 0.7 | 2.3 |
|  | Cabbage | *y*=171923*x*-1035.8 | 0.9995 | 5-500 | 1.27 | 0.6 | 2.0 |
| PCB153 | Hexane | *y=*99835*x+*1.0339 | 0.9999 | 5-500 | - | - | - |
|  | Cucumber | *y*=121018*x*-374.43 | 0.9999 | 5-500 | 1.21 | 0.6 | 1.8 |
|  | Tomato | *y*=109127*x*+72.556 | 0.9999 | 5-500 | 1.09 | 0.8 | 2.5 |
|  | Lettuce | *y*=111846*x*+128.91 | 0.9999 | 5-500 | 1.12 | 0.9 | 3.0 |
|  | Cabbage | *y*=126699*x*-668.60 | 0.9995 | 5-500 | 1.27 | 0.8 | 2.6 |
| PCB105 | Hexane | *y=*138331*x-*35.523 | 0.9999 | 5-500 | - | - | - |
|  | Cucumber | *y*=159897*x*-396.24 | 0.9999 | 5-500 | 1.16 | 0.5 | 1.4 |
|  | Tomato | *y*=148280*x*+228.43 | 0.9999 | 5-500 | 1.07 | 0.6 | 1.9 |
|  | Lettuce | *y*=155063*x*+199.43 | 0.9999 | 5-500 | 1.13 | 0.7 | 2.3 |
|  | Cabbage | *y*=175907*x*-1039.0 | 0.9995 | 5-500 | 1.27 | 0.6 | 2.0 |
| PCB138 | Hexane | *y=*92873*x-*3.3875 | 0.9999 | 5-500 | - | - | - |
|  | Cucumber | *y*=114827*x*-318.15 | 0.9999 | 5-500 | 1.24 | 0.6 | 2.0 |
|  | Tomato | *y*=102550*x*+182.62 | 0.9999 | 5-500 | 1.10 | 0.9 | 2.8 |
|  | Lettuce | *y*=104653*x*+203.98 | 0.9999 | 5-500 | 1.13 | 1.0 | 3.3 |
|  | Cabbage | *y*=118960*x*-518.64 | 0.9995 | 5-500 | 1.28 | 0.9 | 2.9 |
| PCB126 | Hexane | *y=*130504*x-*159.48 | 0.9999 | 5-500 | - | - | - |
|  | Cucumber | *y*=153285*x*-499.18 | 0.9999 | 5～500 | 1.17 | 0.5 | 1.7 |
|  | Tomato | *y*=141101*x*+171.63 | 0.9999 | 5-500 | 1.08 | 0.7 | 2.2 |
|  | Lettuce | *y*=147944*x*+144.08 | 0.9999 | 5-500 | 1.13 | 0.8 | 2.7 |
|  | Cabbage | *y*=168300*x*-1148.0 | 0.9994 | 5-500 | 1.29 | 0.8 | 2.4 |
| PCB167 | Hexane | *y=*112660*x-*1.5376 | 0.9999 | 5-500 | - | - | - |
|  | Cucumber | *y*=134708*x*-353.90 | 0.9999 | 5-500 | 1.20 | 0.6 | 1.7 |
|  | Tomato | *y*=122930*x*+172.78 | 0.9999 | 5-500 | 1.09 | 0.7 | 2.3 |
|  | Lettuce | *y*=127592*x*+197.97 | 0.9999 | 5-500 | 1.14 | 0.9 | 2.9 |
|  | Cabbage | *y*=145035*x*-759.76 | 0.9995 | 5-500 | 1.29 | 0.8 | 2.6 |
| PCB156 | Hexane | *y=*111260*x-*1.7690 | 0.9999 | 5-500 | - | - | - |
|  | Cucumber | *y*=133461*x*-361.07 | 0.9999 | 5-500 | 1.20 | 0.6 | 1.9 |
|  | Tomato | *y*=121745*x*+175.88 | 0.9999 | 5-500 | 1.09 | 0.8 | 2.4 |
|  | Lettuce | *y*=126398*x*+187.00 | 0.9999 | 5-500 | 1.13 | 0.9 | 3.0 |
|  | Cabbage | *y*=143304*x*-782.21 | 0.9995 | 5-500 | 1.19 | 0.8 | 2.6 |
| PCB157 | Hexane | *y=*103225*x+*31.497 | 0.9999 | 5-500 | - | - | - |
|  | Cucumber | *y*=124664*x*-326.05 | 0.9999 | 5-500 | 1.21 | 0.6 | 1.8 |
|  | Tomato | *y*=112980*x*+201.25 | 0.9999 | 5-500 | 1.09 | 0.8 | 2.6 |
|  | Lettuce | *y*=116501*x*+220.70 | 0.9999 | 5-500 | 1.13 | 1.0 | 3.1 |
|  | Cabbage | *y*=132477*x*-671.50 | 0.9995 | 5-500 | 1.28 | 0.9 | 2.7 |
| PCB180 | Hexane | *y=*74064*x+*26.614 | 0.9999 | 5～500 | - | - | - |
|  | Cucumber | *y*=95764*x*-363.54 | 0.9999 | 5-500 | 1.29 | 0.9 | 2.9 |
|  | Tomato | *y*=83257*X* +39.886 | 1.0000 | 5-500 | 1.12 | 1.2 | 3.9 |
|  | Lettuce | *y*=83948*x*+117.46 | 0.9999 | 5-500 | 1.15 | 1.4 | 4.5 |
|  | Cabbage | *y*=95138*x*-441.51 | 0.9994 | 5-500 | 1.28 | 1.2 | 4.0 |
| PCB169 | Hexane | *y=*104880*x-*24.715 | 0.9999 | 5-500 | - | - | - |
|  | Cucumber | *y*=118321*x*-84.107 | 0.9999 | 5-500 | 1.13 | 0.7 | 2.4 |
|  | Tomato | *y*=116437*x*+164.04 | 0.9999 | 5-500 | 1.11 | 1.0 | 3.1 |
|  | Lettuce | *y*=120599*x*+140.26 | 0.9999 | 5-500 | 1.32 | 1.2 | 3.8 |
|  | Cabbage | *y*=138206*x*-869.90 | 0.9994 | 5-500 | 1.32 | 1.1 | 3.5 |
| PCB189 | Hexane | *y=*86112*x+*14.230 | 0.9999 | 5-500 | - | - | - |
|  | Cucumber | *y*=87339*x*+304.84 | 0.9995 | 5-500 | 1.01 | 0.6 | 2.0 |
|  | Tomato | *y*=95716*x*+76.514 | 0.9999 | 5-500 | 1.11 | 0.9 | 2.8 |
|  | Lettuce | *y*= 97415*x*+130.94 | 0.9999 | 5-500 | 1.13 | 1.0 | 3.3 |
|  | Cabbage | *y*=111210*x*-554.18 | 0.9993 | 5-500 | 1.29 | 0.9 | 3.0 |

T_ABLE_ S3: Spiked recoveries and RSDs of the 18 PCBs in the tested vegetable matrices (*n*=6)

| Analyte | Spiked  (μg/kg) | Cucumber | | Tomato | | Lettuce | | Cabbage | |
| --- | --- | --- | --- | --- | --- | --- | --- | --- | --- |
|  |  | Recovery(%) | RSD(%) | Recovery(%) | RSD(%) | Recovery(%) | RSD(%) | Recovery(%) | RSD(%) |
| PCB28 | 5 | 108.3 | 1.8 | 98.8 | 4.4 | 106.4 | 4.5 | 102.6 | 2.9 |
|  | 10 | 99.0 | 4.8 | 105.0 | 3.2 | 96.7 | 5.7 | 90.1 | 7.9 |
|  | 100 | 108.9 | 3.5 | 105.5 | 3.0 | 97.5 | 3.9 | 109.5 | 1.3 |
| PCB52 | 5 | 110.5 | 3.0 | 102.9 | 5.1 | 105.5 | 4.5 | 97.6 | 2.0 |
|  | 10 | 97.3 | 6.1 | 95.2 | 12.7 | 97.5 | 7.2 | 92.5 | 8.8 |
|  | 100 | 108.8 | 3.5 | 105.4 | 3.0 | 97.7 | 4.1 | 109.3 | 0.9 |
| PCB101 | 5 | 110.6 | 2.0 | 97.4 | 3.9 | 105.4 | 4.3 | 101.0 | 2.1 |
|  | 10 | 111.3 | 3.8 | 99.2 | 8.2 | 99.5 | 7.0 | 90.2 | 6.8 |
|  | 100 | 108.9 | 3.8 | 105.5 | 3.5 | 97.8 | 4.2 | 114.9 | 1.1 |
| PCB81 | 5 | 112.2 | 4.3 | 101.6 | 2.8 | 101.3 | 3.4 | 99.8 | 2.6 |
|  | 10 | 97.6 | 2.1 | 103.5 | 5.3 | 103.5 | 5.0 | 93.1 | 9.8 |
|  | 100 | 109.2 | 4.0 | 104.3 | 3.5 | 97.0 | 4.2 | 109.4 | 1.2 |
| PCB77 | 5 | 110.9 | 4.5 | 101.3 | 3.2 | 103.9 | 3.0 | 100.8 | 2.7 |
|  | 10 | 98.0 | 7.4 | 103.1 | 6.1 | 105.5 | 5.6 | 95.3 | 9.8 |
|  | 100 | 110.3 | 3.8 | 104.3 | 3.6 | 97.3 | 4.1 | 113.8 | 1.0 |
| PCB123 | 5 | 111.0 | 2.2 | 99.1 | 4.5 | 107.6 | 2.9 | 102.5 | 2.8 |
|  | 10 | 106.1 | 4.9 | 101.6 | 6.2 | 98.7 | 4.4 | 89.7 | 6.4 |
|  | 100 | 109.7 | 4.4 | 105.4 | 3.3 | 98.8 | 4.4 | 110.5 | 0.6 |
| PCB118 | 5 | 109.8 | 2.0 | 98.3 | 4.0 | 105.0 | 4.1 | 102.0 | 3.3 |
|  | 10 | 103.2 | 4.4 | 100.8 | 5.8 | 97.5 | 6.0 | 89.6 | 7.3 |
|  | 100 | 108.4 | 4.4 | 105.8 | 3.2 | 98.4 | 4.5 | 115.3 | 0.5 |
| PCB114 | 5 | 111.5 | 3.3 | 98.9 | 5.1 | 106.0 | 4.2 | 102.0 | 1.8 |
|  | 10 | 105.3 | 5.0 | 100.4 | 6.4 | 96.4 | 5.7 | 88.5 | 6.8 |
|  | 100 | 108.9 | 4.1 | 105.6 | 3.5 | 97.9 | 4.4 | 110.2 | 0.9 |
| PCB153 | 5 | 109.8 | 5.7 | 88.6 | 3.2 | 88.1 | 4.1 | 104.9 | 6.2 |
|  | 10 | 101.1 | 6.5 | 96.5 | 8.9 | 100.2 | 7.7 | 89.0 | 6.4 |
|  | 100 | 108.8 | 4.1 | 105.7 | 3.5 | 97.3 | 4.3 | 111.0 | 1.3 |
| PCB105 | 5 | 111.0 | 2.0 | 98.3 | 4.8 | 106.2 | 3.6 | 103.2 | 4.0 |
|  | 10 | 105.0 | 5.1 | 101.5 | 6.1 | 98.7 | 5.4 | 89.1 | 7.2 |
|  | 100 | 108.9 | 4.2 | 105.5 | 3.7 | 98.0 | 4.3 | 115.5 | 1.6 |
| PCB138 | 5 | 108.5 | 6.4 | 88.3 | 7.9 | 97.0 | 9.7 | 96.3 | 13.4 |
|  | 10 | 108.0 | 2.6 | 92.6 | 8.0 | 100.2 | 8.0 | 90.3 | 7.9 |
|  | 100 | 108.5 | 4.2 | 105.4 | 3.7 | 85.0 | 14.0 | 111.7 | 0.6 |
| PCB126 | 5 | 113.8 | 3.6 | 99.3 | 4.3 | 106.6 | 4.1 | 103.4 | 7.8 |
|  | 10 | 107.0 | 5.5 | 99.5 | 7.7 | 106.0 | 4.6 | 95.4 | 9.2 |
|  | 100 | 108.8 | 4.5 | 103.9 | 4.3 | 113.9 | 13.1 | 116.5 | 0.8 |
| PCB167 | 5 | 110.1 | 3.6 | 89.9 | 4.6 | 101.7 | 7.0 | 103.6 | 5.0 |
|  | 10 | 107.5 | 4.7 | 98.8 | 7.9 | 99.8 | 5.6 | 89.0 | 6.9 |
|  | 100 | 108.6 | 4.4 | 105.6 | 3.7 | 93.1 | 7.3 | 111.7 | 0.6 |
| PCB156 | 5 | 111.5 | 3.5 | 91.7 | 4.1 | 106.3 | 4.0 | 104.3 | 6.1 |
|  | 10 | 99.0 | 4.4 | 97.3 | 8.2 | 99.2 | 5.3 | 88.5 | 6.6 |
|  | 100 | 108.6 | 4.3 | 105.6 | 3.7 | 97.8 | 4.2 | 112.3 | 0.6 |
| PCB157 | 5 | 110.3 | 3.5 | 92.6 | 4.3 | 107.5 | 5.3 | 100.4 | 3.2 |
|  | 10 | 103.2 | 3.2 | 96.2 | 9.3 | 101.0 | 5.2 | 88.9 | 6.4 |
|  | 100 | 108.7 | 4.5 | 105.8 | 3.6 | 95.6 | 4.8 | 111.9 | 0.9 |
| PCB180 | 5 | 106.7 | 3.6 | 85.7 | 5.5 | 99.2 | 15.6 | 105.1 | 3.1 |
|  | 10 | 99.4 | 4.2 | 93.3 | 11.3 | 103.9 | 13.5 | 89.1 | 6.0 |
|  | 100 | 108.0 | 4.2 | 106.4 | 3.6 | 86.1 | 17.6 | 112.7 | 0.7 |
| PCB169 | 5 | 115.2 | 3.2 | 100.3 | 4.8 | 105.0 | 3.1 | 101.5 | 2.5 |
|  | 10 | 102.9 | 6.2 | 95.4 | 8.8 | 109.1 | 4.5 | 98.0 | 9.6 |
|  | 100 | 109.1 | 4.5 | 106.9 | 4.4 | 100.2 | 4.4 | 110.8 | 0.6 |
| PCB189 | 5 | 108.2 | 2.7 | 84.5 | 2.2 | 110.7 | 3.3 | 107.9 | 2.6 |
|  | 10 | 93.8 | 6.4 | 92.1 | 10.2 | 103.8 | 10.8 | 90.8 | 5.9 |
|  | 100 | 108.3 | 4.6 | 107.8 | 3.6 | 97.9 | 4.1 | 111.6 | 0.8 |
